# Intelligent Optimization and Impact Analysis of Energy Efficiency and Carbon Reduction in the High-Temperature Sintered Ore Production Process

**DOI:** 10.3390/ma17225410

**Published:** 2024-11-05

**Authors:** Yuxing Yuan, Jingchao Sun, Lei Zhang, Su Yan, Tao Du, Hongming Na

**Affiliations:** 1 SEP Key Laboratory of Eco-Industry, Northeastern University, Shenyang 110819, China; 2 Engineering Research Center of Frontier Technologies for Low-Carbon Steelmaking, Ministry of Education, Northeastern University, Shenyang 110819, China; 3 Frontiers Science Center for Industrial Intelligence and Systems Optimization, Northeastern University, Shenyang 110819, China

**Keywords:** sintering process, multi-objective optimization, energy saving, improving efficiency, reducing pollution emissions

## Abstract

The coordinated optimization of energy conservation, efficiency improvement, and pollution reduction in the sintering production process is vital for the efficient and sustainable development of the sintering department. However, previous studies have shown shortcomings in the multi-objective collaborative optimization of sintering systems and the quantification of pollutant impacts. To address these, this paper proposes a multi-objective optimization method integrated with the NSGA-III algorithm and establishes an integrated system optimization model for sintered ore production and high-temperature waste heat recovery. The results demonstrate significant improvements: energy utilization efficiency increased by 0.67%, energy consumption decreased by 17.3 MJ/t, production costs were reduced by 11.45 CNY/t, and the emissions of CO_2_, SO_2_, and NO_x_ were reduced by 0.464 kg/t, 0.034 kg/t, and 0.008 kg/t, respectively. Additionally, the study identified optimal configuration parameters and analyzed the quantitative impact of several key factors on multiple indicators. The results also show that reducing the water content of the mixture, decreasing the middling coal content in the fuel, and increasing the thickness of the material layer are effective strategies to reduce energy consumption and pollutant emissions in the sintering process. Overall, implementing these optimizations can bring significant economic and environmental benefits to the steel industry.

## 1. Introduction

As a typical representative of heavy industry, the steel industry has continually faced challenges such as high energy consumption, significant pollution, and economic fluctuations. In 2022, global steel production reached 1.890 billion tons, with energy consumption per ton of steel exceeding 20.99 GJ [1]. In the complex production chain of steel, sintered ore is the most important raw material in the ironmaking process, and its production scale has exceeded 1.078 billion tons in 2022 [2]. Notably, it is worth stating that the sintering process accounts for the second-largest proportion of energy consumption and carbon emissions in the steel manufacturing process, accounting for 6–10% and 5.7–7.8% [1,2], respectively. Sintering is also the process with the highest pollutant emissions in the entire steel smelting process, with SO_2_, NOx, and particulate matter accounting for 64.84%, 78.72%, and 56.40% of the total emissions in the blast furnace-converter steelmaking route [3,4], respectively. Therefore, optimizing sintering operations and process parameters to achieve high efficiency, energy conservation, environmental protection, and cost reduction has become crucial for enhancing the competitiveness and sustainable development of steel enterprises.

At present, significant progress has been made in energy conservation and pollution reduction in the sintering process within the steel industry. These advancements can be roughly divided into the following categories. Firstly, fuel substitution involves using cleaner and more efficient energy sources [5,6], such as semi-coke, biomass, and natural gas, to replace coke powder and coal [7]. Secondly, efficient combustion and material ratio optimization involve the rational combination of fuel components [8], the optimization of low-carbon batching schemes, and the precise monitoring and control of key parameters. Thirdly, comprehensive treatment of air leakage and air volume control technology for large-scale sintering machines and systems has been adopted, reducing the air leakage rate to below 35% [2]. Fourthly, there is full recovery and use of the waste heat generated during the sintering process [9,10,11]. This involves constructing and optimizing systems to recover waste heat from flue gases and sensible heat from sintered ore [12]. Fifth, flue gas purification and resource recycling have reduced emissions of SO_2_, NO_x_, and particulate matter by nearly 80% and have increased the recycling of iron-containing waste materials [6,13]. These technological advancements have resulted in significant energy savings and a substantial reduction in environmental impact.

Among these advancements, optimization plays a crucial role in industrial process control and technological development. Previous studies have found that optimization research for sintering primarily focuses on two aspects: optimization of the sintering thermal process and optimization of waste heat recovery. In terms of the optimization of the thermal process, Liu et al. [14,15] proposed a sintering ore ratio optimization model based on mathematical programming, which improved the energy-saving and emission reduction effect by 4.92%. Yuan et al. [16] achieved a cost reduction of 8.46% in the overall optimization of the steel manufacturing process while meeting the requirements for ore blending in the production process. In addition, when aiming for a comprehensive carbon ratio in the sintering process, Hu et al. [17] developed an integrated prediction model for the comprehensive coke ratio in multiple modes for real-time predicting and optimizing of carbon efficiency during the process [18]. Chen et al. [19] used backpropagation neural network (BPNN) modeling and the particle swarm optimization (PSO) algorithm to reduce the comprehensive carbon ratio by 2.40 kg/t.

On the other hand, in optimizing the recovery of high-temperature sintered ore waste heat, Feng et al. [20] aimed to maximize the exercise output. They determined the optimal fabric height of the sintered ore layer in the circular cooler, resulting in a 22.80% increase in the exercise output. When optimizing the waste heat recovery and exergy destruction quantity of the sintering vertical cooling furnace, they increased the waste heat recovery rate to 83.02% by determining the optimal structural and operating parameters [21]. Under the optimal temperature parameter combination, the recovery rate of the low-grade sinter cooling flux gas reached 64.86% [22]. Tian et al. [23] quantified the influence of sintering cooler operating parameters on indicators such as waste heat recovery rate and final sintering temperature. They used the Non-Dominated Sorting Genetic Algorithm II (NSGA-II) to solve the multi-objective optimization model and identify the optimal operating conditions. Liu et al. [24] also used NSGA-II to achieve the dual objectives of waste heat utilization and cost for sintered ore cooling beds, and found the optimal operating conditions of air flow rate to be about 400 kg/s, the cooling bed to be 0.8 m to 1.0 m, and the moving speed to be 0.020 m to 0.022 m. Overall, previous studies have mainly optimized processes [25] and key operating parameters [26,27], such as operating speed, flow rate, temperature, time, pressure, and atmosphere, which can effectively improve energy utilization and recovery rates. However, these studies have primarily focused on optimizing either the thermal process or waste heat recovery individually. There is a lack of comprehensive models that integrate the sintering thermal process with waste heat recovery into a unified system. Additionally, there is insufficient collaborative research addressing the interplay between energy efficiency, cost, energy consumption, and pollutants. The analysis of how sintering process operations and parameter optimization affect pollutant emissions is also lacking, particularly concerning the key factors influencing the synergy between energy conservation and pollutant emission in multi-objective integrated optimization. Therefore, this study addresses these gaps by developing a comprehensive model, conducting multi-objective optimization, and analyzing parameter impacts to advance the coordinated improvement of sintering processes in terms of energy conservation, environmental protection, and efficiency enhancement.

Therefore, based on the intricate principles of metallurgical chemistry and the process mechanisms involved in sintered ore formation, this study establishes a comprehensive system model for both sintered ore production and high-temperature waste heat recovery. The Non-Dominated Sorting Genetic Algorithm III (NSGA-III) optimization algorithm is utilized to perform multi-objective optimization targeting energy efficiency, energy consumption, and economic costs in the sintering process. This approach identifies the Pareto front and determines the optimal solutions for balancing quality, energy conservation, and cost objectives. Additionally, it derives the optimal operating conditions for resource allocation and analyzes the quantitative effects of various key factors on multiple objectives and pollutant emissions throughout the production process. This paper is organized as follows. Section 2 provides the explanation of the methodology, Section 3 describes the optimization algorithms and data sources, Section 4 analyzes and discusses the multi-objective optimization results of the sintering plant, including a comparison of evaluation indicators before and after optimization and an examination of various influencing factors, and Section 5 draws conclusions based on the above analysis and discussion.

## 2. Materials and Methods

### 2.1. Research Boundary

**Figure 1:**
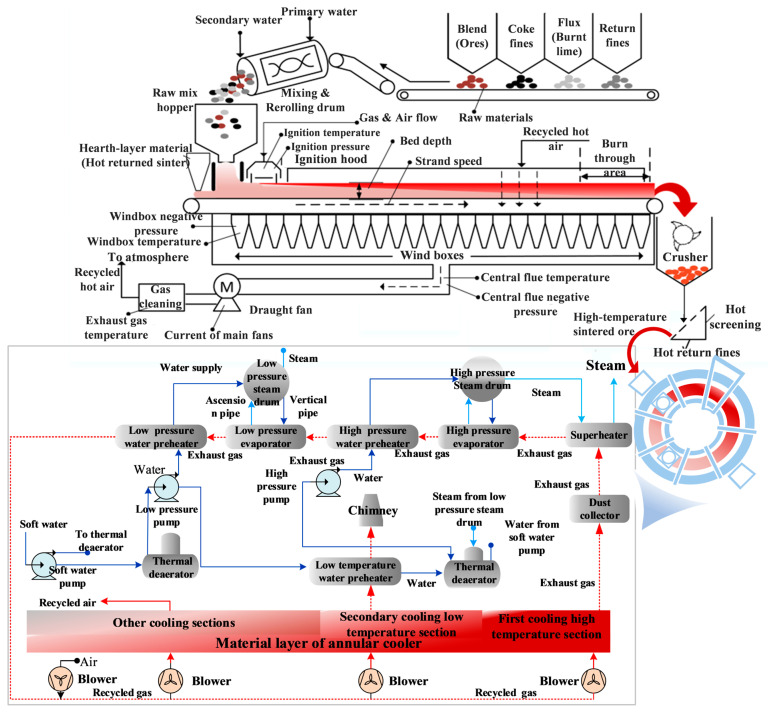
Sintering process and equipment employed in the production cycle at the SP [15,28].

This study takes the sintering plant (SP) of a steel enterprise as the research subject, The specific materials and processes are illustrated in Figure 1, which includes two main components: the high-temperature sintering process and the high-temperature waste heat recovery from sintered ore. The sintering plant involves the combination of ore and auxiliary materials at high temperatures, generated by fuel combustion, to form a solid mass. The equipment used in this process includes silos, mixers, igniters, sintering machines, crushers, fans, and flues. The sensible heat recovery process involves a blower directing cooling air onto the ring cooling bed. This cold air undergoes multi-stage gas-solid heat exchange with the high-temperature sintered ore, absorbing heat from the sintered ore. The heated air is then collected by hoods on top of the sintered ore cooling bed and recycled through various heat exchangers. The equipment used in this process includes ring coolers, hoods, pumps, fans, and heat exchangers.

### 2.2. Model Establishment

The sintering thermal process model involves the coupling of multiple pieces of field information, including mass, heat, momentum, and chemical reactions. Firstly, there is the material conversion process. This process describes the mass energy relationship of SP based on various physical and chemical reaction mechanisms, using the principles of conservation of matter and conservation of energy. The equilibrium relationships of each element in the model can be summarized as follows:

(1) $$\underset{i}{\sum }\left(m_{SP,i,in}\times {\left[X\right]}_{SP,i,in}\right)=\underset{j}{\sum }\left(m_{SP,j,out}\times {\left[X\right]}_{SP,j,out}\right)$$

where the left input item includes various sintering powders, quicklime, limestone, dolomite, return ore, iron slag, coke powder, coal powder, gas, etc. $\left[X\right]$ is the composition of element *X* in the material or energy, and the components such as Fe, FeO, CaO, MgO, SiO_2_, Al_2_O_3_, P, and S in the material have the same equilibrium relationship. The products or by-products of the sintering process include sintered ore, return ore, furnace dust, flue gas, etc.

The ingredients are meticulously produced to meet the specified alkalinity requirements. The required range for the ternary alkalinity of sintered ore is 1.75 to 2.10. The calculation is as follows:

(2) $$R=\frac{\sum_{I}m_{SP,i,in}\times {\left[\mathrm{CaO}\right]}_{SP,i,in}+m_{SP,i,in}\times {\left[\mathrm{MgO}\right]}_{SP,i,in}}{\sum_{I}\left(m_{SP,i,in}\times {\left[{\mathrm{SiO}}_{2}\right]}_{SP,i,in}\right)}$$

Secondly, the energy consumed and released during the material conversion process, along with the flow and loss of energy, are primarily constrained by the first law of thermodynamics. The overall heat balance equation is as follows:

(3) $$\begin{array}{c} Q_{SP,sen,in}+Q_{SP,oxidi,in}+Q_{SP,air,in}+Q_{SP,circul,in}\\ \;\;\;\;\;\;\;\;\;=Q_{SP,sen,out}+Q_{SP,decom,out}+Q_{SP,circul,out}+Q_{SP,gas,out}\\ +Q_{SP,incom,out}+Q_{SP,loss} \end{array}$$

where the input heats are the sensible heat of various materials, the heat released by the oxidation of fuel and iron-containing minerals, and the heat brought in by circulation. The outputs are product and by-product sensible heat, reaction decomposition heat, cycle heat, flue gas heat, incomplete combustion loss, and heat loss.

The calculation formulas for the sensible heat carried by materials and gases are:

(4) $$Q_{m,i}=\int m_{i}\times c_{m,i}\times dt$$

(5) $$Q_{v,i}=\int V_{i}\times c_{v,i}\times dt$$

The reactions in the sintering process include water evaporation and condensation, carbon combustion, carbonate decomposition, iron oxide decomposition, oxidation and reduction reactions, removal of harmful substances such as sulfur and potassium, and consolidation reactions. The main reactions and reaction heats involved in this model are shown in Table 1.

(6) $$Q_{r}=\underset{r}{\sum }\left(\underset{i}{\sum }\left(m_{SP,i,in}\times {\left[X\right]}_{SP,i,in}\right)-\underset{j}{\sum }\left(m_{SP,j,out}\times {\left[X\right]}_{SP,j,out}\right)\right)/M_{r}\times \Delta H_{r}$$

After the sintered ore is flipped, the sensible heat of the sintered ore is recovered by a circular cooler. The circulating hot air, after heat exchange, enters the waste heat boiler to generate steam.

(7) $$Q_{SP,SCR}=a_{air}\cdot \eta_{SCR}\cdot m_{SP,\sin ter,out}\left(\int_{T_{SCR,\sin ter,out}}^{T_{SCR,\sin ter,in}}c_{SP,\sin ter}\left(T\right)dT\right)\;=\left(\sum S_{SP,s,out}\times h_{s}\right)$$

where $a_{air}$ is the comprehensive heat transfer coefficient between sintered ore and air, and $\eta_{SRC}$ is the efficiency of the waste heat boiler of the ring cooler, %. $T_{SRC,\sin ter,in}$, and $T_{SRC,\sin ter,out}$ refer to the temperatures of the sintered ore entering and exiting the sintering ring cooler, respectively, K. Additionally, $S_{SP,s,out}$ and $h_{s}$ are the s-th steam extraction amount and corresponding enthalpy values, kg/t and kJ/kg, respectively.

### 2.3. Evaluation Indexes

#### 2.3.1. Heat Utilization Efficiency (HUE)

The definition and classification of energy efficiency quantitatively represents the effective energy supply level of thermal equipment for specific purposes [29,30], as defined in Equation (1). Energy efficiency is calculated as the ratio of the effective utilization of energy to the energy input during the manufacturing process.

(8) $$\eta_{SP}=\frac{\sum Q_{useful}}{\sum_{i}Q_{i,in}}$$

where $Q_{useful}$ is the useful heat required for the material reaction process, the heat carried by the product, and some by-products, kJ. $\underset{i}{\sum }Q_{i,in}$ is the total energy input, which is the heat brought into the equipment by the material and energy and the heat released by the oxidation reaction, kJ. Effective heat refers to the heat required for the material reaction process.

#### 2.3.2. Energy Consumption (EC)

When studying energy conservation issues in steel enterprises or processes, the primary focus is on the energy consumption per ton of physical production. This is defined as the difference between the energy consumed per unit of the product process and auxiliary production during the statistical period and the energy recovered and supplied externally [31,32]. The specific process of energy consumption is as follows:

(9) $$e_{SP}=\frac{\sum_{i}m_{i,in}\times \varepsilon_{i,in}-\sum_{k}r_{k}\times \varepsilon_{k}}{P}$$

where $e_{SP}$ is the energy consumption of the SP, MJ/t. $m_{i,in}$ is the amount of i-th energy consumption in the SP, kg/t, m^3^/t.

#### 2.3.3. Production Costs (PC)

The production process cost index calculates the production cost of each process and procedure based on the different sources of raw materials, solvents, energy, and power, considering both the purchase price and some internal prices. The production cost of each process product includes material cost, energy cost, maintenance cost, labor cost, depreciation cost, and energy recovery income. The specific details are as follows:

(10) $$C_{cost}=\underset{i}{\sum }\left(c_{i}\times m_{i,in}\right)+\left(c_{main}+c_{labor}+c_{dep}\right)-\underset{k}{\sum }\left(c_{k}\times r_{k}\right)$$

where $C_{cost}$ is the production cost per ton of sintered ore products, CNY/t. $c_{i}$ is the price coefficient of the i-th material or energy, CNY/t/kg, CNY/t/m^3^, or CNY/t/kWh; $c_{main}$, $c_{labor}$, and $c_{dep}$ are the unit product maintenance, labor, and depreciation price coefficients, respectively, CNY/t. $c_{k}$ is the price coefficient for the k-th energy recovered in the process, CNY/t/kg, CNY/t/m^3^.

#### 2.3.4. Pollutants and Carbon Emissions

The accounting of pollutant and carbon emissions in SP is as follows:

(11) $$E_{SO_{2}}=\left(1-\beta_{SO_{2}}\right)\sum \left(m_{SP,i,in}\times {\left[S\right]}_{SP,i,in}-m_{SP,j,out}\times {\left[S\right]}_{SP,j,out}\right)\times O_{SP,i}$$

(12) $$E_{NO_{x}}=\left(1-\beta_{NO_{x}}\right)\underset{I}{\sum }\left(m_{SP,i,in}\times {\left[N\right]}_{SP,i,in}\right)\times \varphi_{SP,NO_{x},i}$$

(13) $$E_{CO_{2}}=\sum \left(m_{SP,i,in}\times {\left[C\right]}_{SP,i,in}-m_{SP,j,out}\times {\left[C\right]}_{SP,j,out}\right)\times \frac{44}{12}\;+\sum \left(m_{SP,i,in}\times f\right)$$

where $O_{SP,i}$ is the oxidation rate of sulfur, while $\beta_{SO_{2}}$ indicates the desulfurization rate of the desulfurization equipment. ${\left[N\right]}_{SP,i,in}$ is the nitrogen content in the fuel. The coefficient for nitrogen oxide generation is denoted as $\varphi_{SP,NO_{x},i}$. Carbon emissions are categorized into direct and indirect emissions. Direct carbon emissions refer to the amount of carbon dioxide directly released from the process, whereas indirect carbon emissions pertain to the emissions resulting from the energy consumed during production. The variable f represents the carbon emission factor.

## 3. Optimization Algorithms and Data Sources

### 3.1. Optimization Algorithm

This paper aims to optimize the heat utilization efficiency, energy consumption, and cost of the sintering process. The mathematical formulation of the multi-objective optimization problem is described as follows:

(14) $$Min\left(and\;Max\right)\;y=\left[f_{1}\left(x\right),f_{2}\left(x\right),\cdot \cdot \cdot ,f_{n}\;\left(x\right)\right]\left(n=1,\;2,\ldots ,N\right)\;st.h\left(x\right)=\left[h_{1}\left(x\right),h_{2}\left(x\right),..h_{m}\left(x\right)\right]=0,m=1,2,\ldots M\;g\left(x\right)=\left[g_{1}\left(x\right),g_{2}\left(x\right),..g_{d}\left(x\right)\right]\leq 0,d=1,2,\ldots D\;x=\left[x_{1},\;x_{2},\ldots ,x_{i},\ldots x_{I}\right]\;x^{l}\leq x\leq x^{u}$$

where y is the objective function, and *N* is the total number of optimization objectives. $f_{n}\;\left(x\right)$ is the n-th sub objective function. *g*(*x*) and *h*(*x*) represent the D-term inequality and M-term equality constraint conditions, respectively. These constraints define the feasible domain. Additionally, $x^{l}$ and $x^{u}$ are the lower and upper limits of the parameters, respectively. The optimization objective of this paper is to maximize heat utilization efficiency while minimizing energy consumption and carbon emissions during the sintering process. The various constraints in the optimization model include product quality and composition, process temperature, air volume requirements, and oxygen content.

In addition, NSGA-III is employed to solve multi-objective optimization problems by introducing a set of predefined reference points generated using the Das-Dennis method [33,34]. These reference points are evenly distributed within the objective space, guiding the population to evenly cover the entire Pareto front. NSGA-III utilizes non-dominated sorting and reference point-based selection mechanisms to maintain population diversity effectively and prevent the solution set from becoming overly concentrated in certain regions of the Pareto front [35,36]. This approach ensures a well-distributed set of optimal solutions across the entire Pareto front, enhancing the robustness and comprehensiveness of the optimization process. Model building and optimization work using Python language editing and 11th Gen Intel (R) Core (TM) i7-11700 @ 2.50 GHz 2.50 GHz processor, 128 GB memory server.

### 3.2. Data Sources

The primary data for this study are sourced from an advanced integrated steel plant located in Tangshan, Hebei Province, China. The plant is equipped with state-of-the-art sintering machines and circular cooling units, and utilizes a dual pressure waste heat boiler to capture low-temperature flue gas. With an annual output of approximately 11 million tons, the plant boasts a product qualification rate of 99% and a drum strength exceeding 81.0%. The main material components of the sintering plant as of August 2022 are detailed in Table A1, Table A2, Table A3 and Table A4 of Appendix A.

### 3.3. Model Validation

In order to ensure the model’s accuracy, on-site statistical data from the case enterprise were used for validation. The comparison results between the field data and the model’s operation are shown in Table 2. The small errors observed are within acceptable limits, indicating that the model is effective and applicable. Additionally, the model’s accuracy was further verified by comparing it with previous research results, as shown in Table 3.

## 4. Results and Discussion

### 4.1. Multi Objective Optimization Results

**Figure 2:**
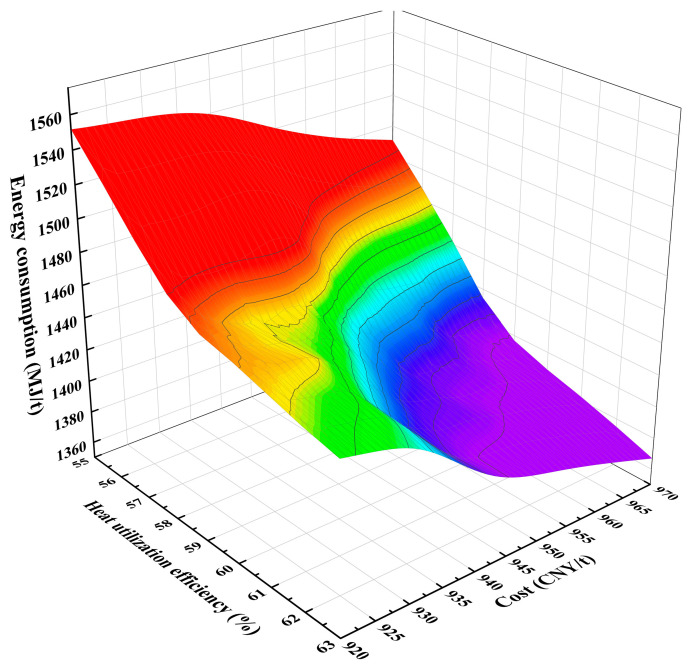
Pareto front of multi-objective optimization results.

This paper is based on the established integrated model of the high-temperature sintering process and high-temperature waste heat recovery in sintering plants. Using the NSGA-III algorithm, we optimized the heat utilization efficiency, energy consumption, and production cost of the sintering model, resulting in a solution set comprising 61 distinct solutions. These solutions illustrate the trade-off relationships between the various objectives, forming a Pareto front. Figure 2 provides a three-dimensional visualization, intuitively showcasing the distribution of the solution set and the interrelationships among the objectives. These solution sets offer a range of choices for sintering decision-makers. Sintering managers typically focus on cost objectives when optimizing the ratio of various ores. To further select multi-objective optimal solutions that prioritize cost, solutions with weights of 0.2, 0.3, and 0.5 for heat utilization efficiency, energy consumption, and production cost objectives, respectively, were chosen from the frontier solution set to identify the optimal solution for the sintering production process. The results before and after optimization are shown in Table 4.

The results showed that the optimized sintering process increased heat utilization efficiency by 0.67%, indicating more efficient energy conversion and utilization under the same production conditions, thereby reducing waste. Energy consumption decreased by 17.3 MJ/t, a reduction of 1.17%, effectively lowering energy use in the production process. Production costs were reduced by 11.45 CNY/t, a decrease of 1.22%. Additionally, CO_2_, and SO_2_ emissions per ton of sintered ore decreased by 0.464 kg/t and 0.034 kg/t, and NOx emissions decreased by 0.008 kg/t. This not only cuts production costs but also reduces pollutants and carbon emissions, contributing positively to environmental protection. The company currently produces over 34,900 tons of sintered ore per day, with an annual output of approximately 11.74 million tons. This production can reduce energy consumption by 203.12 million MJ, production costs by 134.47 million CNY, CO_2_ emissions by 5.45 million kg, SO_2_ emissions by 0.399 million kg, and NO_X_ by 0.094 million kg annually. In summary, the model method proposed in this article effectively optimizes the sintering process. Implementing these optimizations enhances heat utilization efficiency, reduces production costs, and minimizes environmental pollution, while also providing significant economic and environmental benefits to the steel industry.

### 4.2. Parameter and Energy Changes

**Figure 3:**
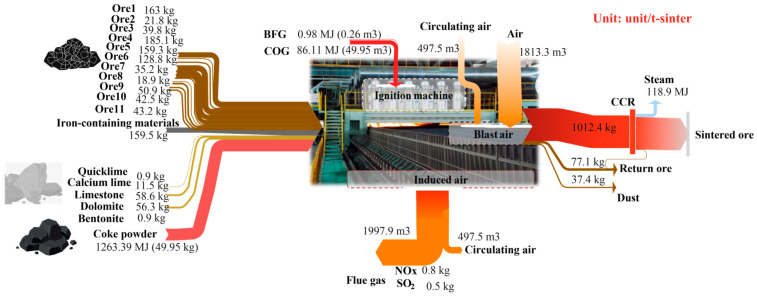
The material and energy parameters of the SP before optimization.

**Figure 4:**
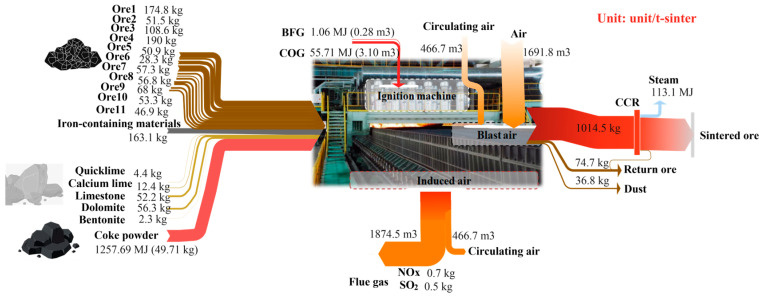
The material and energy parameters of the SP after optimization.

Various ores and fluxes produce high-temperature sintering through oxidation, reduction, and solidification reactions in the action of fuel, producing exhaust gases containing carbon dioxide, sulfur dioxide, and nitrogen oxides. The material and energy input/output and conversion relationships of various sintering equipment and subsystems, both before and after multi-objective optimization, are illustrated in Figure 3 and Figure 4. These figures provide a detailed display of the flow direction and conversion process of materials and energy. Through comparing different ore ratios, it was discovered that adjusting the mix led to a decrease in the cost of sintered ore products from 942.38 CNY to 930.93 CNY per ton. The highest unit price of ore6 decreased from 128.81 kg/t to 28.26 kg/t, while the lower unit price of ore3 increased from 39.78 kg/t to 108.58 kg/t. This change significantly impacted sintering costs and resulted in a substantial difference in iron content. This was achieved by incorporating better iron ore powders such as ore2, ore8, and ore9, with increases of 29.71 kg/t, 22.17 kg/t, and 37.85 kg/t, respectively. Therefore, through multi-objective optimization under strict production constraints, the ore ratio can be adjusted more effectively to reduce sintering costs in a scientifically sound manner.

**Figure 5:**
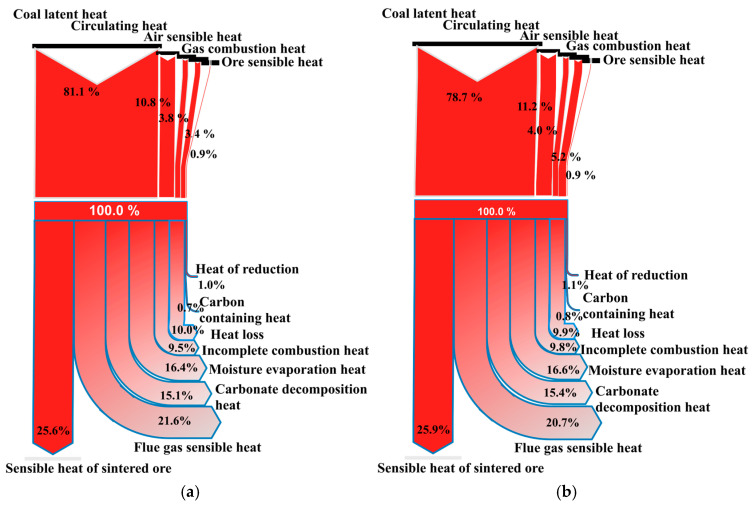
Heat utilization input and output before (**a**) and after (**b**) optimization of sintering high-temperature process.

In addition, the amount of air injected during the high-temperature operation of sintering is determined by a comprehensive analysis of factors such as the theoretical air volume needed for fuel combustion, air leakage, circulating air volume, and the limitation of oxygen content in the flue gas. From Figure 3 and Figure 4, it can be clearly seen that after optimization, the air volume injected into the sintering process decreased from 1813.26 m^3^/t to 1691.78 m^3^/t, and the flue gas volume decreased from 1997.89 m^3^/t to 1874.45 m^3^/t. As illustrated in Figure 5a,b the optimization of the sintering high-temperature process significantly impacted the proportion of heat input and output. Notably, the reduction in flue gas volume led to a decrease in the proportion of heat loss carried away by the flue gas, reducing it from 21.56% to 20.73%. The reduction in air volume and the decrease in heat loss carried away by flue gas after optimization are the primary reasons for the improvement in heat utilization efficiency in the sintering process. This optimization also resulted in decreased energy consumption, as reflected by a 0.45% reduction in coke powder consumption. Additionally, although the circulating hot air volume in the sintering internal and external circulation decreased from 497 m^3^/t before optimization to 466.74 m^3^/t, the utilization efficiency of circulating heat slightly reduced. This slight reduction is also attributable to the decrease in the total exhaust air volume.

The comparison of the proportion of heat input and output before and after optimization is shown in Figure 5. Due to the adjustment of the ore type ratio after optimization, the proportion of sensible heat conduction, moisture evaporation heat, and carbonate decomposition heat of sintered ore increased by 0.3%, 0.2%, and 0.3%, respectively. This improvement is due to the optimization of multiple ore ratios, in which increases the grade of the mixed ores and sintered ore. This optimization leads to a slight reduction in the amount of gangue and impurities introduced by the material, thereby decreasing the energy consumption required for gangue melting and discharge. Consequently, more heat is used for effective utilization in the production process. Additionally, the reduction in exhaust volume decreases the proportion of exhaust heat loss by 0.83%. Overall, the optimization of parameters such as ore blending and airflow in the sintering process, has led to a reduction in the cost and energy consumption of the sintering process, as well as an improvement in heat utilization efficiency. It has also reduced the number of pollutants and carbon emissions in the exhaust gas during the sintering process.

### 4.3. Analysis of Influencing Factors

**Figure 6:**
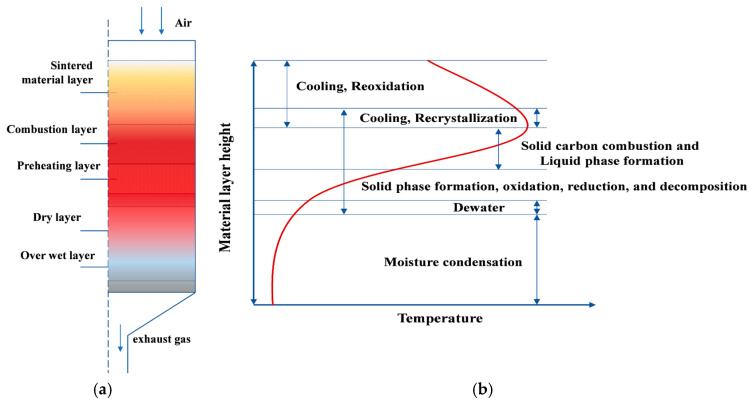
(**a**) Distribution diagram of material layer in SP. (**b**) Temperature changes and reaction processes of each material layer in SP [37].

Based on the in-depth analysis results in the previous section, we have identified the significant impact of raw material parameters and operating parameters on determining the quality, production efficiency, energy utilization efficiency, and emission characteristics of sintered ore. To provide more systematic guidance for sintering production practice and optimize process control, this section combines the basic principles of material layer distribution, temperature gradient, and reaction interval in the sintering thermal process (as shown in Figure 6a,b), and analyzes in detail the specific impact laws of raw material chemical composition (iron ore grade, impurity content), and key operating parameters (material layer thickness, mixture moisture content, and fuel ratio) on improving multiple key sintering indicators.

#### 4.3.1. Changes in Sintered Ore Grade

**Figure 7:**
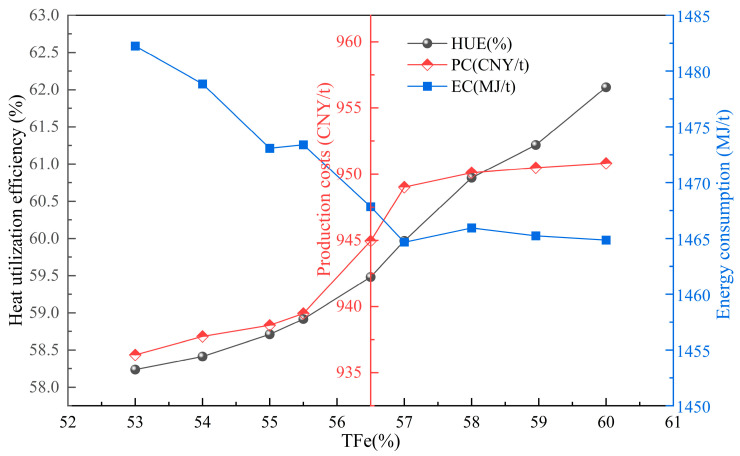
The influence of changes in iron content in sintered ore on sintering heat utilization, energy consumption, and cost.

**Figure 8:**
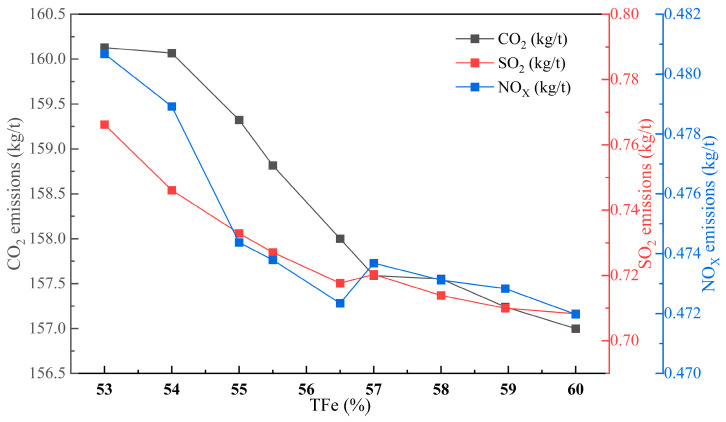
The influence of changes in iron content in sintered ore on pollutant emissions and carbon emissions in sintering plants.

The quantitative relationship between the change in sintered ore grade and sintering indicators is illustrated in Figure 7. The sintered ore grade has a positive correlation with heat utilization efficiency and production cost of the sintering production process. For every 1% increase in sintered ore grade, the heat utilization efficiency and production cost decrease by 0.81% and 0.19%, respectively. However, the sintering energy consumption increases by 0.15%. The impact of changes in sintered ore grade on sintering pollutants is depicted in Figure 8. As the grade increases, sintering pollutants and carbon emissions are significantly reduced. CO_2_, SO_2_, and NO_x_ emissions are reduced by 3.13 kg/t, 0.058 kg/t, and 0.009 kg/t, respectively. This reduction is mainly due to changes in ore powder. Higher ore grades contain fewer impurities, leading to reduced heat consumption during the sintering production process and decreased fuel consumption. Therefore, appropriately increasing the sintered ore grade is beneficial for efficient sintering energy conservation, pollution reduction, and high efficiency.

#### 4.3.2. Changes in the Thickness of the Material Layer

**Figure 9:**
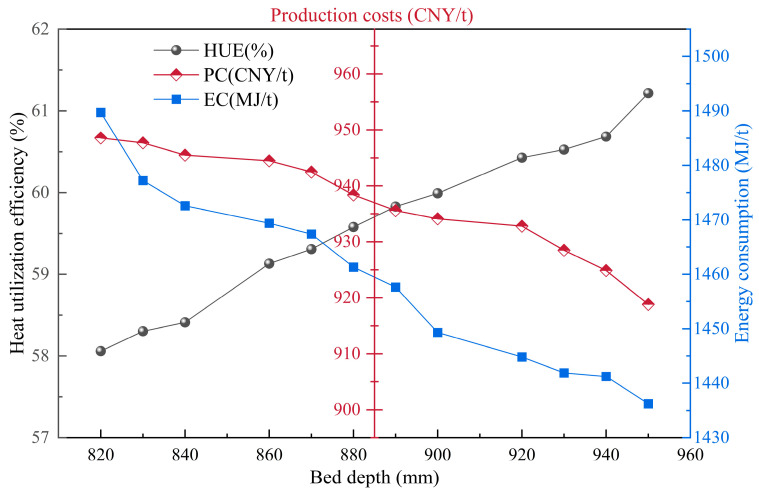
The influence of material layer thickness on sintering energy utilization, energy consumption, and carbon emissions.

**Figure 10:**
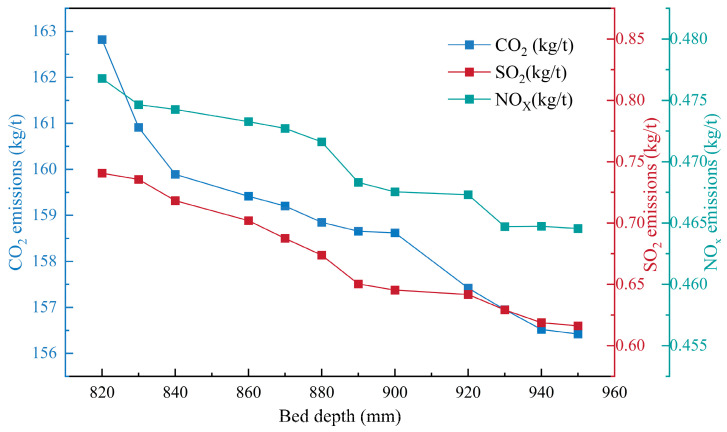
The influence of material layer thickness on sintering pollutants and carbon emissions.

By increasing the thickness of the material layer, the energy utilization of the sintering process can be promoted, fully utilizing the self-heat storage effect of the material layer and improving the heat supply of the lower material layer [38,39]. Based on the constructed model, this section briefly discusses and quantifies the impact of material layer thickness changes on various sintering indicators under the condition that technology meets practical production needs. As shown in Figure 9 and Figure 10, the increase in the material layer can greatly improve heat utilization efficiency. When the material layer changes from 820 mm to 950 mm, the production cost and energy consumption decrease by 3.13% and 3.59%, respectively, showing a linear relationship. This is mainly due to the increase in output and the effect of thermal recycling. At the same time, SO_2_, NO_X_, and CO_2_ emissions decrease by more than 2.5%. Overall, increasing the thickness of the sintering material layer is very beneficial for the sustainable development of sintering.

#### 4.3.3. Change in Coal Ratio

**Figure 11:**
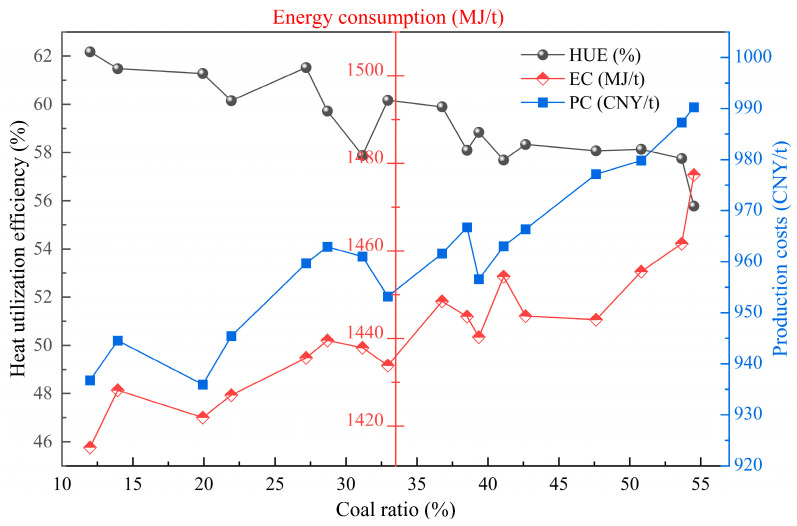
The influence of changes in coal ratio in fuel on sintering heat utilization, energy consumption, and cost.

**Figure 12:**
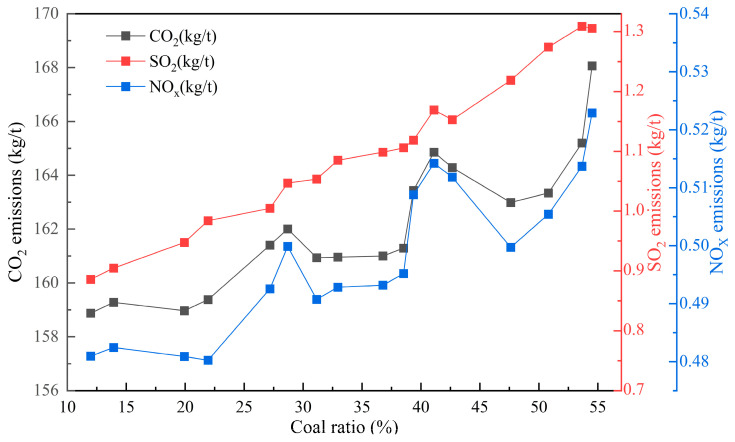
The influence of changes in coal ratio in fuel on pollutant emissions and carbon emissions in sintering plants.

Figure 11 and Figure 12 show the quantitative relationship between the change in the proportion of middling coal consumption in the total fuel consumption and the impact on the sintering index. Based on the energy content of the fuel, varying dosage ratios of coal and coke powder are applied. As the proportion of coal increases from 10% to 54%, the cost price of coke powder is lower than the market price of coal, but the calorific value is higher than that of coal. As the coal consumption increases, the production cost and energy consumption of sintering gradually increase. At the same time, with the production of the same product, as the amount of coal used increases, it inevitably leads to an increase in the total amount of coal and coke powder used, as well as an increase in the sulfur content and nitrogen-containing substances in coal, especially the sulfur content. As a result, the emissions of pollutants and carbon during the sintering process increase, with CO_2_, SO_2_, and NO_x_ emissions increasing by 5.82 kg/t, 0.432 kg/t, and 0.031 kg/t, respectively, which is not conducive to energy conservation and pollution reduction in the sintering process. Compared to purchased coal, the coking process has already removed most impurities from coal. Therefore, using coke powder instead of coal in the sintering process offers several advantages.

#### 4.3.4. Changes in Moisture Content of Materials

**Figure 13:**
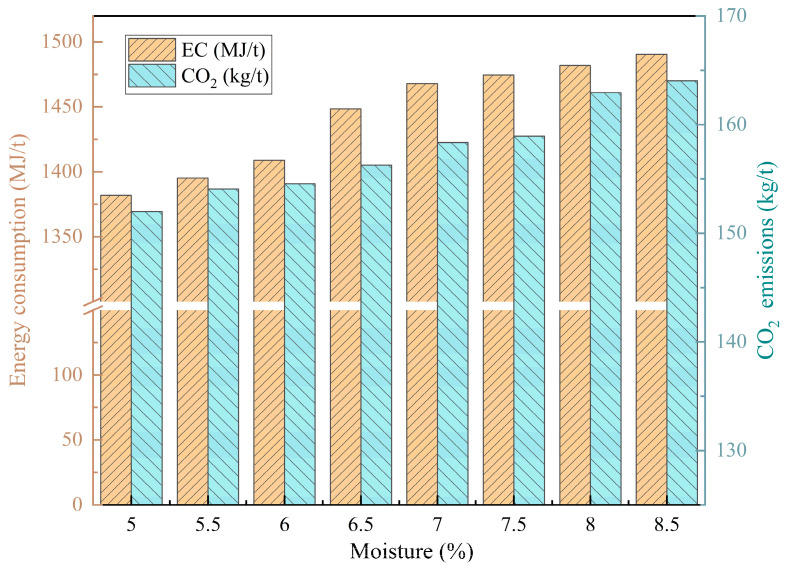
Influence of material moisture content on sintering energy consumption and carbon emissions.

The moisture content of the material is shown in Figure 13. The change in moisture content directly affects the energy consumption and carbon emissions of the sintering process. As the moisture content increases from 5% to 8.5%, the energy consumption and carbon emissions gradually increase. This is because more water evaporation requires excessive heat consumption, which needs to be supplemented by an increase in fuel consumption. Therefore, energy consumption and carbon emissions increase significantly, by 7.84% and 7.91%, respectively, as a result of using coke powder in the sintering process; other pollutant emissions remain unchanged. Therefore, accurately and strictly controlling material moisture can effectively reduce energy consumption in the sintering process.

## 5. Conclusions

This paper is grounded in the complex principles and mechanisms of metallurgical chemistry related to sintered ore formation. Firstly, an integrated system model for sintered ore production and high-temperature waste heat recovery was established. A multi-objective optimization method for the sintering process, combined with the NSGA-III optimization algorithm, was then proposed. This method optimizes the heat utilization efficiency, energy consumption, and production cost of the sintering process. Additionally, it quantifies the influence of several typical factors on various sintering indicators and pollutant emissions. The research has led to the following conclusions:

(1) Using the NSGA-III algorithm, a Pareto front solution set for the multi-objective optimization of the sintering process was obtained, and the optimal solution for balancing quality, energy saving, and cost objectives was found. After optimization, the heat utilization efficiency of the sintering process increased by 0.67%, energy consumption decreased by 17.3 MJ/t, and production costs decreased by 11.45 CNY/t. Additionally, CO_2_ and SO_2_ emissions per ton of sintered ore decreased by 0.464 kg/t and 0.034 kg/t, and NOx emissions decreased by 0.008 kg/t. This optimization effectively improved the heat utilization efficiency of the sintering process while reducing energy consumption, production costs, pollutants, and carbon emissions.
(2) In addition, optimized operating parameters for the optimal allocation of production resources, such as ore blending, flux, fuel consumption, and various airflow parameters, were obtained. These optimizations are beneficial for enhancing energy savings, improving efficiency, and reducing pollution in the sintering process. By implementing these optimizations, enterprises are expected to reduce energy consumption by 203.12 million MJ, production costs by 134.47 million CNY, CO_2_ emissions by 5.45 million kg, SO_2_ emissions by 0.399 million kg, and NO_X_ by 0.094 million kg annually. It has brought significant economic and environmental benefits to the steel industry.
(3) By studying and quantifying the influence of various key factors on multiple targets and pollutant emissions in the production process, especially changes in parameters such as sintered ore grade, material layer thickness, mixed material moisture content, and coal ratio, it was found that for every 1% increase in sintered ore grade, heat utilization efficiency improved by 0.81%, energy consumption decreased by 0.19%, and sintering production cost increased by 0.15%. Additionally, while ensuring production quality requirements are met, reasonably reducing the moisture content in the mixture and minimizing the consumption of coal in the fuel, along with increasing the material layer thickness, can contribute to lowering sintering energy consumption and reducing pollutant emissions.

## Figures and Tables

**Table 1:** The main chemical reactions and reaction enthalpy reactions in SP.

Reaction Types	Reaction	ΔH/kJ·mol^−1^
Oxidation reaction	C + 0.5O_2_ = CO	−110.5
	C + O_2_ = CO_2_	−393.5
	2Fe_3_O_4_ + 0.5O_2_ = 3Fe_2_O_3_	−235.8
	3FeO + 0.5O_2_ = 3Fe_3_O_4_	−302.4
	4FeS_2_ + 8O_2_ = 2Fe_2_O_3_ + 6SO_2_	−3310
Reduction reaction	CO_2_ + 2C = 2CO	+172.5
	3Fe_2_O_3_ + CO = 2Fe_3_O_4_+ CO_2_	−47.2
	Fe_3_O_4_ + CO = 3FeO + CO_2_	+19.4
Decomposition reaction	MeCO_3_ = MeO + CO_2_	FeCO_3_(+85.1), CaCO_3_(+178.6), MgCO_3_(+120.9)
	MeSO_4_ = MeO + SO_2_+ 0.5O_2_	FeSO_4_(+340.4), CaSO_4_(+500.6), MgSO_4_(+221.6)

**Table 2:** Comparison of the simulated results and field data.

Category	Type	Field Data	Simulated Data
Input materials	Mixture materials (kg/t)	1176.030	1176.030
	iron ore (kg/t)	888.378	888.378
	Returned ore (kg/t)	159.457	159.457
	Flux (kg/t)	128.691	128.691
	Coke powder (kg/t)	49.952	49.952
	COG (m^3^/t)	4.78	4.78
Output materials	Tfe (%)	56.902	56.994
	FeO of sintered ore (%)	8.590	8.500
	C of sintered ore (%)	0.059	0.043
	CaO of sintered ore (%)	10.100	10.492
	MgO of sintered ore (%)	1.784	2.107
	SiO_2_ of sintered ore (%)	4.934	5.512
	Al_2_O_3_ of sintered ore (%)	1.851	2.006
	TiO_2_ of sintered ore (%)	0.108	0.071
	P of sintered ore (%)	0.044	0.057
	S of sintered ore (%)	0.025	0.004
	Recovery of steam (kg/t)	39.62	39.19

**Table 3:** Comparison of the simulated and previous research results.

Index	Field Data	Simulated	Wu et al. [8]	Sun et al. [32]
Heat utilization efficiency	57.83%	57.98%	57.92%	/
Energy consumption	1474.44 MJ	1464.76 MJ	1471.8 MJ	1402 MJ

**Table 4:** Comparison of results before and after optimization.

Index	HUE (%)	EC (MJ/t)	PC (CNY/t)	CO_2_(kg/t)	SO_2_(kg/t)	NOx (kg/t)
Before optimization	57.83	1474.44	942.38	160.040	0.781	0.484
After optimization	58.50	1457.14	930.93	159.576	0.747	0.476

## Data Availability

The original contributions presented in the study are included in the article, further inquiries can be directed to the corresponding authors.

## Abbreviations

SP	Sintering plant	COG	Coke Oven Gas
SRC	Sintering ring cooler	BFG	Blast Furnace Gas
HUE	Heat utilization efficiency	NSGA-III	Non-dominated Sorting Genetic Algorithm III
EC	Energy consumption	CNY	Chinese Yuan
PC	Production costs		
i	The i-th type of material or energy	$T_{SRC,\sin ter,in}$	Temperature of sintered ore entering the ring cooler, °C
j	The j-th type of output material or energy	$T_{SRC,\sin ter,out}$	Temperature of the sintered ore exiting the ring cooler, °C
$m_{SP,i,in}$	The input dosage of the i-th material or energy, kg/t	$S_{SP,s,out}$	Extraction volume of the s-th type of steam, kg/t
$m_{SP,j,out}$	The output dosage of the j-th material or energy, kg/t	$h_{s}$	Enthalpy value of the s-th type of steam, kJ/kg
R	Dosage of material in process, kg/t	$e_{SP}$	energy consumption of the sintering process, MJ/t
$Q_{SP,i,in}$	The i-th type of input heat in SP, kJ/t	$r_{k}$	The dosage of k-th energy recovered and supplied externally, kg/t, m^3^/t
$Q_{SP,j,out}$	The j-th type of output heat in SP, kJ/t	$\varepsilon_{i,in}$, $\varepsilon_{k}$	Conversion coefficient of standard coal, MJ/t, MJ/m^3^
$m_{i}$	The dosage of the i-th material or energy, kg/t	P	The dosage of sintered ore produced during the statistical period, kg/t
$c_{m,i}$	The specific heat capacity of the i-th material or energy, kJ/(kg·K)	$C_{cost}$	production cost per ton of sintered ore products, CNY/t
$V_{i}$	The specific heat capacity of the i-th gas, m^3^/t	$c_{i}$	Price coefficient of the i-th substance or energy, CNY/kg, CNY/m^3^, CNY/kWh
$c_{v,i}$	Specific heat capacity of the i-th gas, kJ/(m^3^·K)	$c_{main}$, $c_{labor}$, $c_{dep}$	Price coefficients for maintenance, labor, and depreciation of unit products, CNY/t
r	The r-th chemical reaction	$c_{k}$	Price coefficient for the k-th type of energy recovery, CNY/kg, CNY/m^3^
$M_{r}$	Relative molecular mass of the r-th material	$O_{SP,i}$	Oxidation rate of sulfur, %
$\Delta H_{r}$	Reaction enthalpy of the r-th reaction, kJ/mol	$\beta_{SO_{2}}$	The desulfurization rate of desulfurization equipment, %
$a_{air}$	Comprehensive heat transfer coefficient between sintered ore and air, W/(m²·K)	$\varphi_{SP,NO_{x},i}$	Nitrogen oxide generation coefficient
$\eta_{SRC}$	Efficiency of waste heat boiler in sintering ring cooler, %	f	Carbon emission factor, kg/t, kg/m^3^, kg/kWh

## Appendix A

**Table A1:** The composition and price parameters of raw material in sintering plant.

Material Species	Component (%)									Price (CNY)
	TFe	FeO	SiO_2_	CaO	MgO	Al_2_O_3_	S	P	TiO_2_	
Ore1	65.80	0.23	1.42	0.45	0.01	1.22	0.01	0.06	0.09	830.00
Ore2	62.50	0.22	4.20	0.48	0.03	2.30	0.02	0.09	0.10	815.00
Ore3	61.60	0.31	4.35	0.45	0.03	1.88	0.04	0.05	0.11	500.00
Ore4	58.50	0.23	4.16	0.44	0.01	1.42	0.01	0.05	0.09	795.00
Ore5	56.20	0.20	5.53	0.10	0.03	2.22	0.03	0.08	0.11	760.00
Ore6	66.60	26.00	2.50	0.60	0.40	1.30	0.11	0.01	0.17	870.00
Ore7	60.60	11.32	10.33	0.69	0.83	1.14	0.02	0.02	0.00	800.00
Ore8	66.03	26.01	2.08	0.55	1.04	0.90	0.19	0.02	0.00	840.00
Ore9	57.43	0.28	5.57	0.24	0.64	1.20	0.02	0.07	0.06	720.00
Ore10	58.74	0.91	5.16	0.01	0.10	2.61	0.02	0.05	0.00	730.00
Ore11	59.61	0.33	5.51	0.27	0.12	2.81	0.01	0.05	0.00	750.00
Quicklime	0.00	0.00	4.75	56.95	6.25	1.49	0.09	0.00	0.00	680.00
Limestone	0.00	0.00	0.84	51.14	3.01	0.93	0.02	0.00	0.00	500.00
Dolomite	0.00	0.00	1.83	31.09	20.29	0.00	0.02	0.00	0.00	180.00
Bentonite	1.18	0.00	68.40	1.51	2.48	13.95	0.00	0.00	0.08	300.00
Calciumlime	0.00	0.00	0.64	83.96	3.20	0.33	0.01	0.00	0.00	880.00

**Table A2:** Proportion of gas components in sintering plant.

Gas	H_2_	O_2_	N_2_	CO	CO_2_	CH_4_	C_2_H_6_	C_3_H_8_	C_2_H_4_	C_2_H_2_
COG	59.8	0.14	3.54	6.53	1.86	23.60	0.62	0.00	1.35	0.00
BFG	4.535	0.25	49.60	24.86	20.75	0.01	0.00	0.00	0.00	0.00

**Table A3:** The proportion of each component of fuel coal in sintering plant.

Coal Species	Volatile Content $\left(V_{\mathrm{daf}}/\%\right)$	Carbon Content (Car/%)	Sulfur Content $\left(S_{\mathrm{td}}/\%\right)$	Ash Content $\left(A_{d}/\%\right)$
Anthracite	9.45	78.92	0.14	12.50
Coke	0.008752	85.77	0.59	11.95

**Table A4:** Composition requirements for sintered ore products in sintering plant.

Composition	TFe	FeO	SiO_2_	CaO	MgO	Al_2_O_3_	S	P	TiO_2_
Sintered ore	55~58	≤9.00	≤5.80	≤11.00	≤2.50	≤2.20	≤0.03	≤0.09	≤0.25

**Table A5:** The composition of sintered ore before and after optimization.

Composition	TFe	FeO	SiO_2_	CaO	MgO	Al_2_O_3_	S	P	TiO_2_
Before	56.32	8.50	5.75	10.95	2.42	2.09	0.0043	0.0016	0.084
After	56.68	8.40	5.66	10.94	2.47	2.02	0.0041	0.0011	0.068
